# Clusters of Adolescent and Young Adult Thyroid Cancer in Florida Counties

**DOI:** 10.1155/2014/832573

**Published:** 2014-04-28

**Authors:** Raid Amin, James J. Burns

**Affiliations:** ^1^Department of Mathematics and Statistics, University of West Florida, Pensacola, FL 32514, USA; ^2^Florida State University College of Medicine, P.O. Box 33655, Pensacola, FL 32508, USA

## Abstract

*Background*. Thyroid cancer is a common cancer in adolescents and young adults ranking 4th in frequency. Thyroid cancer has captured the interest of epidemiologists because of its strong association to environmental factors. The goal of this study is to identify thyroid cancer clusters in Florida for the period 2000–2008. This will guide further discovery of potential risk factors within areas of the cluster compared to areas not in cluster. *Methods*. Thyroid cancer cases for ages 15–39 were obtained from the Florida Cancer Data System. Next, using the purely spatial Poisson analysis function in SaTScan, the geographic distribution of thyroid cancer cases by county was assessed for clusters. The reference population was obtained from the Census Bureau 2010, which enabled controlling for population age, sex, and race. *Results*. Two statistically significant clusters of thyroid cancer clusters were found in Florida: one in southern Florida (SF) (relative risk of 1.26; *P* value of <0.001) and the other in northwestern Florida (NWF) (relative risk of 1.71; *P* value of 0.012). These clusters persisted after controlling for demographics including sex, age, race. *Conclusion*. In summary, we found evidence of thyroid cancer clustering in South Florida and North West Florida for adolescents and young adult.

## 1. Introduction

Nearly 70,000 adolescents and young adults (AYAs; ages 15–39) are diagnosed with cancer annually in the United States [1]. The AYA population is a special age group that has not been recognized in research investigating cancer etiology and therapy. This age group has failed to show the significant improvements in mortality reported for a wide variety of cancer types in other age categories. Also there is a unique distribution of cancer diagnoses in the AYA population [2]. In the United States thyroid cancer incidence has been growing at a faster pace than other cancers from 1996–2005 [3, 4]. In the AYA population thyroid cancer is fourth in overall frequency with an estimated 28% of new thyroid cancers occurring in this age group [5, 6]. Florida has had significant increases in rates of thyroid cancer [7].

Geographical analyses of thyroid cancer have been conducted in regions around the world where across all continents there are increasing trends [8]. Higher rates were found in districts of Sao Paulo, Brazil, where higher levels of atmospheric particulate matter were measured [9]. In Italy, using SaTScan to detect clusters, iodine deficient mountainous regions had higher rates of thyroid cancer deaths. Space-time cluster analysis showed improvement with iodine prophylaxis in certain regions [10]. Also in Italy, a case-control study showed residence for more than 20 years in regions with higher rates of iodine deficient goiter had higher rates of thyroid cancer [11]. In Cordoba, Argentina clusters of thyroid cancer were found in two regions using SaTScan [12]. In Wisconsin, where there has been a doubling of thyroid cancer rates, geographic areas with higher access to health care and socioeconomic status had higher rates of thyroid cancer raising concerns about medical use of radiation for medical procedures [13]. In a study conducted in Nebraska, the geographic pattern of thyroid cancer correlated with susceptibility of regional ground water to toxins such as atrazine, alachlor, uranium, and gross alpha particle radiation [14]. In England and Wales, high rates were seen in the northern and middle regions of Wales with twice the risk as more southern regions [15]. In Caledonia where there is ten times the rate of other developed countries, there was a sharp increase in rates that remains unexplained [16]. In France over the time period 1975 to 2004, there has been a dramatic increase in thyroid cancer rates with five-fold increases [17].

The main environmental risk factor for thyroid cancer reported in the literature is exposure of the thyroid gland to radiation. Potential sources that have been studied include radiation that comes from the atmosphere, ground water, energy plants, and medical procedures [14, 18–23]. It appears that younger age increases susceptibility to the effects of radiation on the thyroid gland. These effects may not manifest themselves for a prolonged latency period of time after exposure [19, 22]. Some studies as noted above assumed that the increase over time was caused by the use of radiation in medical settings [24, 25]. However, other studies suggest that the trend was associated with other sources of exposure such as atmospheric nuclear fallout [18, 26–28]. The National Academy of Sciences has recently issued a multiphase study to evaluate the risk of cancer in populations near nuclear facilities [29].

Other studies suggest that exposure to chemicals used in the leather, wood, and paper making process and exposure to solvents, pesticides [30], and cancer chemotherapy [31] may also play a role in pathogenesis. Polybrominated diphenyl ethers (PBDEs) that are increasingly being used in plastics, flame retardants, and construction have been shown to have disrupting effect on thyroid function and have been hypothesized to cause thyroid cancer [8, 32].

Still others have suggested that increased diagnostic capabilities have been the major contributor to the observed increased thyroid cancer rates [23, 33].

Study of the environment for cancer risk factors is important to conduct especially in the case of thyroid cancer. Specifically finding a spatial cluster that is defined as unusual rates of disease in a certain geographic area can help generate hypotheses that may eventually lead to causative agents [34–37]. From a public health perspective, geographical cluster analysis is useful to detect areas of excess risk due to a known risk factor. Clusters can be detected either spatially or over time periods through a number of computerized statistical program packages [34, 38, 39]. Spatial research into geographically based cancer risk factors can be challenging given the mobility of populations and long latency from environmental exposures and development of disease [40]. Thus these studies are more hypothesis generating rather than definitive in identification of etiology for cancer.

State cancer registries can provide researchers with a wealth of information regarding cancer rates by demographic variables in specific geographic regions over periods of time. Collecting data since 1981, the Florida Cancer Data System (FCDS) is Florida's statewide, population-based cancer registry [41]. The State of Florida Department of Health, the National Program of Cancer Registries (NPCR) of the Centers for Disease Control and Prevention (CDC), and the Sylvester Comprehensive Cancer Center at the University of Miami Miller School of Medicine support the FCDS. Data from such registries can readily be input into spatial and time cluster computer programs for analysis.

There are studies in the literature that have attempted to link spatially based risk factors and cancer [42–44]. Recent research has found spatial and temporal clusters of leukemia in Ohio [39] and leukemia and brain cancer in Florida [45]. The National Academy of Sciences is planning to conduct research on proximity to nuclear power plants and cancer [29].

In this study, we sought to identify thyroid cancer clusters in the AYA population in Florida.

## 2. Methods

### 2.1. Study Area and Population

The raw incidence rates of thyroid cancer for the age group 15–39 years (adolescents and young adults-AYA), broken down by age group, sex, and race, were downloaded in January 2010 from the FCDS website, while the corresponding annual population counts by county for age, sex, and race were downloaded from the Census Bureau website [46].

Analysis was conducted for the 67 counties in Florida. We planned to remove Union County if found to be in cluster of thyroid cancer, because it is where the Florida's state correctional system Medical Center is located [45].

Geographic county center points (centroids) were used to represent each county for the SaTScan [34] analysis. These were calculated and projected on maps in the geographical information system ArcGIS [47]. The study population included the entire population of adolescent and young adults 15–39 years of age in the state of Florida during the time period 2000–2008.

### 2.2. Data Sources

The data for this study was obtained from the Florida Cancer Data System (FCDS), a publicly available website [41]. The FCDS is Florida's cancer statewide cancer registry created by the Florida Department of Health in 1978. In this registry, cancer types are classified according to the International Classification of Diseases for Oncology Third Edition (ICD-O-3) [48] by the FCDS. The International Classification of Diseases, Tenth Revision (ICD-10), is used to code cancer deaths, and the International Classification of Diseases, Ninth Revision, Clinical Modification (ICD-9-CM), is used for classification of diagnoses in hospitals [49]. FCDS uses rules for coding specified in the Surveillance, Epidemiology and End Results (SEER) Multiple Primary and Histology Coding Rules, National Institutes of Health, 2007 [50]. The initial dataset was downloaded in January 2011 and then combined with population data for use for a cluster analysis. The Florida population data, including demographics such as sex, age, and race, was obtained from the Census Bureau for years 2000–2008. For each county, we obtained the total population at risk, stratified by age, sex, and race.

### 2.3. Data Analysis

The software package SaTScan was the main analysis tool. The incidence counts in each county were used for a purely spatial analysis. Additionally, the time dimension was incorporated for space-time analyses. The assumption for this study is that the incidence of cancer in each county should be distributed according to a Poisson model and that the age-adjusted risk of cancer incidence is the same for all counties controlling for age, sex, and race.

The spatial scan statistics in SaTScan identifies clusters by utilizing a moving window that scans over a map, including different sets of neighboring counties. Multiple window scans of the sixty-seven counties are performed. For each window, the purely spatial scan statistic test found in SaTScan tests the null hypothesis that there is no increased risk versus the alternative that there is a cluster.

As per the SaTScan manual:

“under the Poisson assumption, the likelihood function for a specific window is proportional to

(1) $$\begin{array}{c} {\left(\frac{n}{E}\right)}^{n}{\left(\frac{N-n}{N-E}\right)}^{N-n}I\;\left(n>E\right), \end{array}$$

*N* is the total number of incidences in Florida; *n* is the observed number of cancer cases within the scan window; *E* is the expected number of cancer cases under the null hypothesis. *I* is an indicator function: *I* = 1 when the scan window has a larger number of cancer incidences than expected if the null hypothesis was true, and 0 otherwise. It can be shown that, for a given *N* and *E*, the likelihood increases as the number of incidences, *n*, increases in the scan window”.

How the spatial scan statistic within SaTScan actually identifies cancer clusters is described at http://www.satscan.org in detail [34].

In SaTScan, cluster stability is attained by Monte Carlo simulation with 999 random replications of the data set being created. It is possible to control for multiple demographic confounding variables using the Monte Carlo's test. The output from SaTScan provides a summary of the location of the “most likely clusters” with a *P* value indicating the statistical significance of these clusters. Additionally a more precise estimate for the *P* value is possible in SaTScan by utilizing a Gumbel approximation which is both more efficient and provides higher powered analyses when compared to the Monte Carlo method [51].

SaTScan uses either circular or elliptical windows in its scanning methods to identify clusters. In this study, the circular window option was chosen for the analysis.

Also, in addition to spatial clusters, the data was analyzed for space-time clusters using SaTScan, where a higher rate of disease is searched in a specific spatial region over a discreet time period. For confirmation of space-time clusters, the nonparametric permutation test for space-time analysis was performed in SaTScan. This space-time permutation scan statistic looks for space-time interaction clusters, adjusting for any purely spatial clusters as well as for any temporal trends.

Finally, in this study another program, FleXScan, which can detect irregularly shaped clusters, was utilized to confirm the spatial analysis [36, 37, 52]. A recent study concluded that FleXScan was better at detecting rare diseases in large geographic areas [53].

## 3. Results

**Figure 1:**
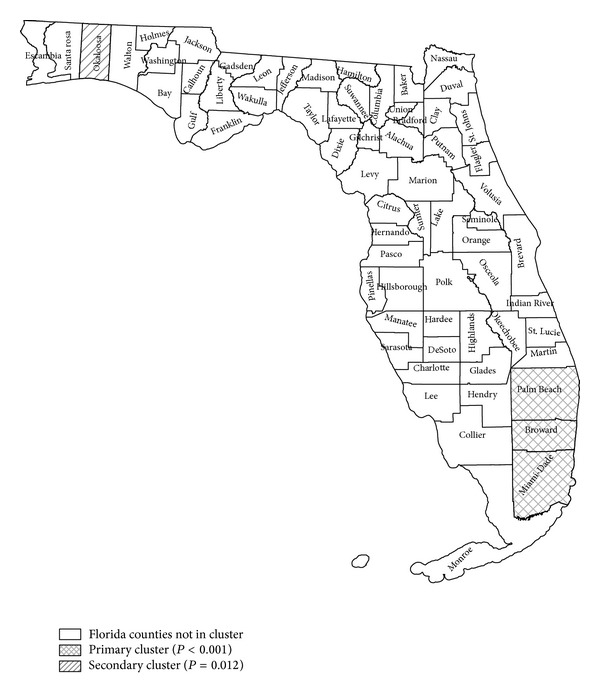
SaTScan purely spatial Poisson analysis for clustering using county data adjusting for age, sex, race as covariates based on data from Florida Cancer Data System (FCDS) cancer registry for thyroid cancer in adolescents and young adults (AYA) 2000–2008. Primary cluster is shown in crosshatched label and contains the South Florida (SF) counties of Miami-Dade, Broward, and Palm-Beach (relative risk 1.26; *P* value < 0.001). Secondary cluster is shown in simple hatched label and contains the northwest (NWF) county of Okaloosa (relative risk 1.71; *P* value = 0.012).

The SaTScan purely spatial analysis revealed two statistically significant thyroid cancer clusters in the state, one in southern Florida (SF) and the other in northwestern Florida (NWF) (see Figure 1 and Table 1). A total of 3,526 AYA cases of thyroid cancer were identified in Florida with incidence rate of 7.3 average annual cases per 100,000. In SF there were 1,300 observed AYA cases of thyroid cancer and 1,118 expected cases, with a relative risk of 1.26, implying that, compared with the state, there is a statistically significant 26% increased risk of AYA thyroid cancer (*P* value <0.001). The probability that the identified SF cluster is random is very small. In the NWF cluster, there were 65 observed case and 38 expected cases, with a relative risk of 1.71 (*P* value of 0.012), which implies that compared with the state of Florida AYA population, in this area there was 1.71 times the risk of being diagnosed with AYA thyroid cancer, a 71% increased risk.

**Figure 2:**
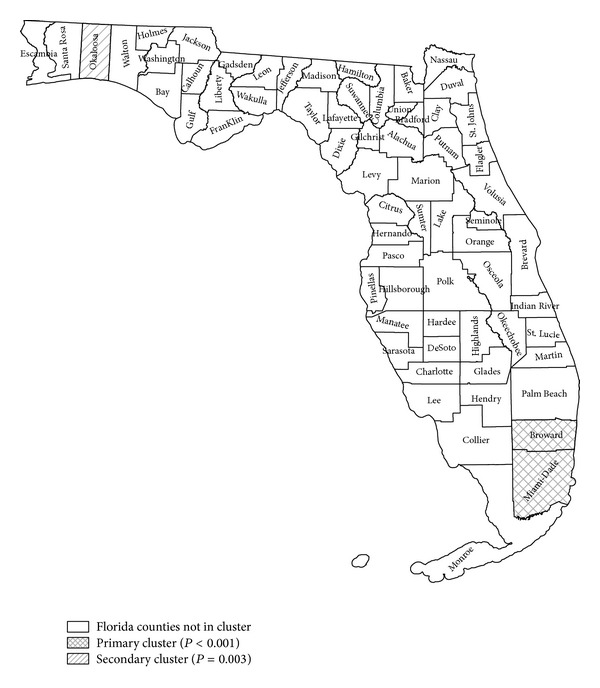
SaTScan purely spatial Poisson analysis for females, adjusted for age and race, for thyroid cancer in adolescents and young adults (AYA) 2000–2008. Primary cluster is shown in crosshatched label and contains the South Florida (SF) counties of Miami-Dade and Broward (relative risk = 1.25, *P* value < 0.001). Secondary cluster is shown in simple hatched label and contains the northwest (NWF) county of Okaloosa (relative risk 1.88, *P* value = 0.003).

A separate analysis was run adjusted for age and sex but not for race. Identical clusters were identified as in the case when rates are adjusted for age, sex, and race. We conclude that race does not play an important role in creating the significant clusters. It is, however, also known that thyroid cancer in females has risen dramatically compared to males. So therefore further analysis was performed utilizing SatScan to determine if any spatial or space-time influences were detectable when analyzing the sexes separately. While the purely spatial analysis for thyroid rates (adjusted for age, sex, and race) resulted in two significant clusters (SF and NWF), when analyzing the data separately by sex, the female data set (adjusted for age and race) (Figure 2) identifies a smaller area of SF as the most likely cluster (*P* value = 0.001, RR = 1.25) and NWF as the secondary cluster (*P* value = 0.003, RR = 1.86). The purely spatial analysis of the male data also reveals a smaller but still separate part of SF as the most likely cluster (*P* value = 0.005, RR = 1.82). No other significant cluster exists for males.

**Figure 3:**
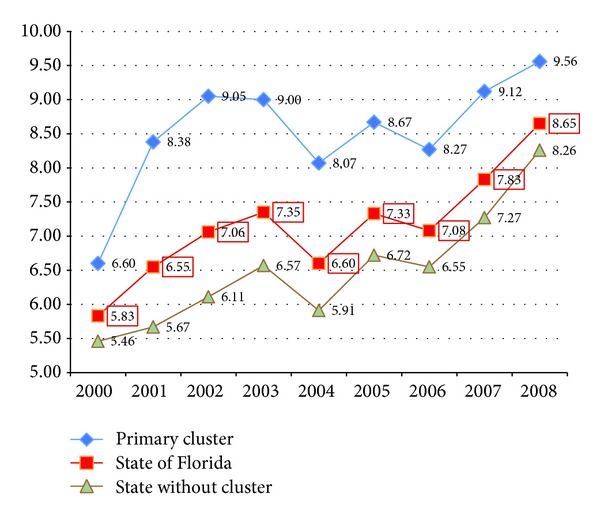
Increasing overall incidence of thyroid cancer in AYA 2000–2008.

The above purely spatial analysis does not include time in the analysis. In order to provide information whether there was a specific time when the cluster occurred, the space-time function in SaTScan was employed. A statistically significant space-time cluster of thyroid cancer was found in SF from 2001 to 2003. The space-time permutation scan statistic which looks for space-time interaction clusters, adjusting for any purely spatial clusters as well as for any temporal trends, however, did* not* identify a surge in any given year for the period 2000–2008. Thus the observed space-time regional cluster seems to be due to an overall statewide increase in the thyroid cancer incidence rates, and thus the space-time findings are artifacts rather than true clusters. A time plot of age adjusted thyroid cancer incidence rates for 2000–2008 also supports the space-time permutation analysis with an increase in thyroid cancer cases statewide over time (Figure 3).

**Figure 4:**
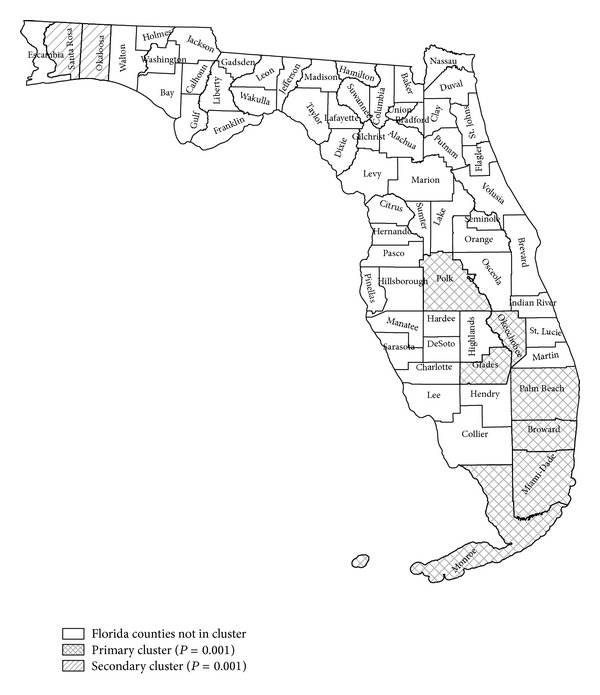
FleXScan purely spatial analysis for thyroid cancer in adolescents and young adults (AYA) 2000–2008. Primary cluster is shown in crosshatched label and contains seven South Florida (SF) counties including Miami-Dade, Palm Beach, Broward, Okeechobee, Monroe, Polk, and Glades (relative risk = 1.14, *P* value = 0.001). Secondary cluster found in Santa Rosa and Okaloosa counties (relative risk 1.77, *P* value = 0.001). Note that this differs from SaTScan analysis because the scanning window in FleXScan can vary in shape, whereas SaTScan is either circular or elliptical.

The results with FleXScan differed slightly from those by SaTScan by including several counties in the “most likely cluster” that were not identified by SaTScan. In addition to Palm Beach, Broward, and Miami-Dade, FleXScan included the counties Okeechobee, Glades, Polk, and Martin in the cluster (Figure 4). FleXScan was not confined to circular shaped clusters, and the position of Lake Okeechobee in the southern part of Florida made it better to also use FleXScan. A secondary cluster was found in Santa Rosa and Okaloosa counties.

## 4. Discussion

This study identified AYA thyroid cancer clusters in SF and the NWF regions. These cancer clusters persisted after controlling for age, sex, and race. With regards to sex specific clusters, in addition to significant increase in cancer rates observed in female and male populations together, taken separately, there were similar spatial regions identified in SF. Also, there is a relative increase in AYA thyroid cancer crude incidence rate in SF during the years 2000–2008. Therefore, the geographic location of residence was found to be relevant to risk of thyroid cancer.

This study provides additional information on cancer risk recently reported in Miami-Dade and Broward counties [54]. This may be due to environmental factors or common risk factors in the areas. Further investigation is needed to identify the potential risk factors in the observed thyroid cancer clusters in these areas. These may include some of the above identified risk variables including exposure to chemical or radiation. Also of interest is the general increase in statewide thyroid cancer. This requires further study.

This study utilized the statistical software (SaTScan) that has been used in analysis of other disease clusters [34]. Identification of clusters of disease in space and time can be complicated by several procedural inaccuracies [55, 56]. Some studies have indicated inconsistencies in results between the different statistical packages [39, 53]. In this study we used both SaTScan and FleXScan with similar results obtained.

Also, in some cases, these studies can be limited by low statistical power [57]. The large sample of cases in this study drawn from AYA thyroid cancer database with 1,300 cases observed in the SF cluster should ensure a sufficiently high statistical power (Table 1).

Another potential problem that can lead to artifactual clustering is misclassification or selection bias due to inaccurate or incomplete case data. Because cancer data from a highly reliable source (FCDS) was used, this problem is minimized. The accuracy of case ascertainment in FCDS has been described and validated elsewhere [7, 58]. FCDS data quality control is excellent and includes routine inspection for duplications and accurate categorization of cancer by demographic factors.

Another problem includes the influence of confounding demographic variables on the analysis. This study is controlled for the effects of these and still observed a statistically significant relative increase in SF compared with the state of Florida. Finally, it is possible there are other unknown confounding variables that may explain these clusters [59].

In summary, we found evidence of spatial clustering of thyroid cancer cases for AYA age range in SF and NWF. This evidence may indicate environmental risk factors influencing these results, predisposing adolescents and young adults in these cluster regions to increased risk of thyroid cancer. Further study is needed to investigate the possible factors contributing to the elevated AYA thyroid cancer rates found.

## Figures and Tables

**Table 1:** SaTScan output 2000–2008 AYA thyroid cancer purely spatial analysis adjusted for age, sex, and race.

	State	Primary cluster	Secondary cluster
AYA population	5,358,013	1,695,088	58,637
Number of cases	3,526	1,300	65
Annual cases per 100,000	7.3	8.5	12.4
Relative risk		1.26	1.71
Log likelihood ratio		21.04	7.78
*P* value		<0.001	0.012

## References

[1] National Cancer Institute Adolescents and young adults (AYA) with cancer Secondary Adolescents and young adults (AYA) with cancer. http://www.cancer.gov/cancertopics/aya.

[2] Bleyer A (2007). Young adult oncology: the patients and their survival challenges. *CA: A Cancer Journal for Clinicians*.

[3] Ries LAG, Melbert D, Krapcho M SEER cancer statistics review, 1975–2005. Secondary SEER cancer statistics review, 1975–2005. http://seer.cancer.gov/csr/1975_2005.

[4] Yu G, Li J, Branovan D, McCormick S, Schantz SP (2010). Thyroid cancer incidence and survival in the national cancer institute surveillance, epidemiology, and end results race/ethnicity groups. *Thyroid*.

[5] UCSW Group United States Cancer Statistics: 1999–2004 incidence and mortality web based report. Secondary United States Cancer Statistics: 1999–2004 incidence and mortality web based report. http://www.cdc.gov/uscs.

[6] National Cancer Institute, National Cancer Institute (2006). Thyroid cancer. *SEER AYA Monograph*.

[7] Mulla ZD, Margo CE (2000). Primary malignancies of the thyroid: epidemiologic analysis of the Florida Cancer Data System Registry. *Annals of Epidemiology*.

[8] Kilfoy B, Zheng T, Holford T (2009). International patterns and trends in thyroid cancer incidence, 1973–2002. *Cancer Causes and Control*.

[9] Yanagi Y (2012). The impact of atmospheric particulate matter on cancer incidence and mortality in the city of São Paulo, Brazil. *Cadernos de Saúde Pública*.

[10] Minelli G, Conti S, Manno V, Olivieri A, Ascoli V (2013). The geographical pattern of thyroid cancer mortality between 1980 and 2009 in Italy. *Thyroid*.

[11] Franceschi S, Fassina A, Talamini R (1989). Risk factors for thyroid cancer in northern Italy. *International Journal of Epidemiology*.

[12] Agost L, Pujol C, Bertone C, Álvarez M, Fantin M Analysis of cancer incidence by department in the province of Cordoba-Argentina (2004-2008): rates of incidence and detection of spatial clusters.

[13] Sprague B, Andersen S, Trentham-Dietz A (2008). Thyroid cancer incidence and socioeconomic indicators of health care access. *Cancer Causes & Control*.

[14] Vanosdel N Spatial patterns of thyroid cancer in Nebraska: exploring possible environmental relationships.

[15] dos Santos Silva I, Swerdlow AJ (1993). Thyroid cancer epidemiology in England and Wales: time trends and geographical distribution. *British Journal of Cancer*.

[16] Truong T, Rougier T, Dubourdieu D (2007). Time trends and geographic variations for thyroid cancer in New Caledonia, a very high incidence area (1985–1999). *European Journal of Cancer Prevention*.

[17] Colonna M, Bossard N, Guizard A, Remontet L, Grosclaude P (2010). Descriptive epidemiology of thyroid cancer in France: incidence, mortality and survival. *Annales d’Endocrinologie*.

[18] Takahashi K, Schoemaker MJ, Trott KR (2003). The relationship of thyroid cancer with radiation exposure from nuclear weapon testing in the Marshall Islands. *Journal of Epidemiology/Japan Epidemiological Association*.

[19] Boice JD (1996). Cancer following irradiation in childhood and adolescence. *Medical and Pediatric Oncology Supplement*.

[20] Shibata Y, Yamashita S, Masyakin VB, Panasyuk GD, Nagataki S (2001). 15 years after chernobyl: new evidence of thyroid cancer. *The Lancet*.

[21] Cardis E, Kesminiene A, Ivanov V (2005). Risk of thyroid cancer after exposure to 131I in childhood. *Journal of the National Cancer Institute*.

[22] Acharya S, Sarafoglou K, LaQuaglia M (2003). Thyroid neoplasms after therapeutic radiation for malignancies during childhood or adolescence. *Cancer*.

[23] Schonfeld S, Lee C, Gonzalez B (2011). Medical exposure to radiation and thyroid cancer. *Clinical Oncology*.

[24] Pottern LM, Stone BJ, Day NE (1980). Thyroid cancer in Connecticut, 1935–1975: an analysis by cell type. *American Journal of Epidemiology*.

[25] Weiss W (1979). Changing incidence of thyroid cancer. *Journal of the National Cancer Institute*.

[26] Kazakov VS, Demidchik EP, Astakhova LN (1992). Thyroid cancer after Chernobyl. *Nature*.

[27] Kaatsch P, Spix D, Schulze-Rath R, Schmiedel S, Blettner M (2008). Leukaemia in young children living in the vicinity of German nuclear power plants. *International Journal of Cancer*.

[28] Fairlie I (2009). Commentary: childhood cancer near nuclear power stations. *Environmental Health*.

[29] National Academy of Sciences (2012). *Analysis of Cancer Risks in Populations Near Nuclear Facilities: Phase I*.

[30] Leux C, Guenel P (2010). Risk factors of thyroid tumors: role of environmental and occupational exposures to chemical pollutants. *Revue d’Epidemiologie et de Sante Publique*.

[31] Veiga L, Bhatti P, Ronckers CM (2012). Chemotherapy and thyroid cancer risk: a report from the childhood cancer survivor study. *Cancer Epidemiology Biomarkers & Prevention*.

[32] Zhang Y, Guo G, Han X (2008). Do polybrominated diphenyl ethers (PBDE) increase the risk of thyroid cancer?. *Bioscience Hypotheses*.

[33] Davies L, Welch HG (2006). Increasing incidence of thyroid cancer in the United States, 1973–2002. *The Journal of the American Medical Association*.

[34] Kulldorff M SaTScan v. 8.0: Software for the spatial and space-time scan statistics. Secondary SaTScan v. 8.0: Software for the spatial and space-time scan statistics. http://www.SaTScan.org/.

[35] Kulldorff M, Heffernan R, Hartman J, Assunção R, Mostashari F (2005). A space-time permutation scan statistic for disease outbreak detection. *PLoS Medicine*.

[36] Takahashi K, Kulldorff M, Tango T, Yih K (2008). A flexibly shaped space-time scan statistic for disease outbreak detection and monitoring. *International Journal of Health Geographics*.

[37] Tango T, Takahashi K (2005). A flexibly shaped spatial scan statistic for detecting clusters. *International Journal of Health Geographics*.

[38] Lawson AB (2006). *Statistical Methods in Spatial Epidemiology*.

[39] Wheeler D (2007). A comparison of spatial clustering and cluster detection techniques for childhood leukemia incidence in Ohio, 1996–2003. *International Journal of Health Geographics*.

[40] Jacquez GM, Meliker J, Kaufmann A (2007). In search of induction and latency periods: space-time interaction accounting for residential mobility, risk factors and covariates. *International Journal of Health Geographics*.

[41] University of Miami School of Medicine Florida Cancer Data System: FCDS. Secondary Florida Cancer Data System: FCDS. http://fcds.med.miami.edu.

[42] Caldwell GG (1990). Twenty-two years of cancer cluster investigations at the Centers for Disease Control. *American Journal of Epidemiology*.

[43] Warner SC, Aldrich TE (1988). The status of cancer cluster investigations undertaken by state health departments. *American Journal of Public Health*.

[44] Kulldorff M, Athas WF, Feuer EJ, Miller BA, Key CR (1998). Evaluating cluster alarms: a space-time scan statistic and brain cancer in Los Alamos, New Mexico. *American Journal of Public Health*.

[45] Amin R, Bohnert A, Holmes L, Rajasekaran A, Assanasen C (2010). Epidemiologic mapping of Florida childhood cancer clusters. *Pediatric Blood & Cancer*.

[46] United States Census Bureau United States Census 2010. Secondary United States Census 2010. http://www.census.gov/2010census/.

[47] ArcGIS Version 9.3 program.

[48] Percy C, van Holten V, Muir C (1990). *International Classification of Disease for Oncology*.

[49] World Health Organization (2004). *International Statistical Classification of Diseases and Health Related Problems*.

[50] Howlader N, Noone AM, Krapcho M SEER Cancer Statistics Review. Secondary SEER Cancer Statistics Review. http://seer.cancer.gov/csr/1975_2008.

[51] Abrams A, Kleinman K, Kulldorff M (2010). Approximating p-values for the spatial scan statistic using the Gumbel distribution. *International Journal of Health Geographics*.

[52] Takahashi K, Yokoyama T, Tango T FleXScan v 3.1: Software for the Flexible Scan Statistic.

[53] Goujon-Bellec S, Demoury C, Guyot-Goubin A, Hémon D, Clavel J (2011). Detection of clusters of a rare disease over a large territory: performance of cluster detection methods. *International Journal of Health Geographics*.

[54] Kearney G (2008). A procedure for detecting childhood cancer clusters near hazardous waste sites in Florida. *Journal of Environmental Health*.

[55] Kingsley B, Schmeichel KL, Rubin C (2007). An update on cancer cluster activities at the Centers for Disease Control and Prevention. *Environmental Health Perspectives*.

[56] Wartenberg D, Greenberg M (1993). Solving the cluster puzzle: clues to follow and pitfalls to avoid. *Statistics in Medicine*.

[57] Wartenberg D, Greenberg M (1990). Detecting disease clusters: the importance of statistical power. *American Journal of Epidemiology*.

[58] Howe HL, Lehnherr M (1997). *Cancer Incidence in North America, 1989–1993*.

[59] Thun MJ, Sinks T (2004). Understanding cancer clusters. *CA: A Cancer Journal for Clinicians*.

